# Low-Temperature Defect Healing in the Layered Zintl Phase Li_2_ZnSi

**DOI:** 10.1021/acs.inorgchem.5c05692

**Published:** 2026-01-27

**Authors:** Xian-Juan Feng, Matej Bobnar, Alim Ormeci, Mohammad Mehmandoust, Marcus Schmidt, Bodo Böhme, Mitja Krnel, Michael Baitinger, Julia Maria Hübner

**Affiliations:** † Institute of Nonferrous Metallurgy and Purest Materials, 26545 TU Bergakademie Freiberg, Leipziger Straße 34, 09599 Freiberg, Germany; ‡ Krka, d. d., Šmarješka cesta 6, 8000 Novo mesto, Slovenija; § 28270 Max Planck Institute for Chemical Physics of Solids, Nöthnitzer Strasse 40, 01187 Dresden, Germany; ∥ Fakultät für Chemie und Lebensmittelchemie, 9169 Technische Universität Dresden, Bergstraße 66, 01069 Dresden, Germany

## Abstract

Li_2_ZnSi is a layered Zintl phase composed of heterographene-like Zn–Si sheets separated by Li atoms. Although the intrinsic crystal structure is fully ordered, mechanical handling readily introduces stacking faults of the Zn–Si layers. These defects significantly broaden the ^7^Li and ^29^Si NMR signals and are described by statistically disordered structure models in single-crystal X-ray diffraction. Upon moderate heating to only 310–370 K, the ^7^Li NMR spectra sharpen, while single-crystal X-ray diffraction reveals a fully ordered structure model. The heat-capacity data exhibit a broad endothermic feature during heating, characteristic of a stress-relief annealing process rather than a thermodynamic phase transition. Mechanical treatment strongly affects physical properties, and the transport response in impedance measurements is dominated by grain-boundary effects. Density-functional calculations show that the stacking-fault formation is energetically unfavorable but localized, explaining why the defects are readily introduced mechanically and can be healed at unexpectedly low temperatures.

## Graphical abstract

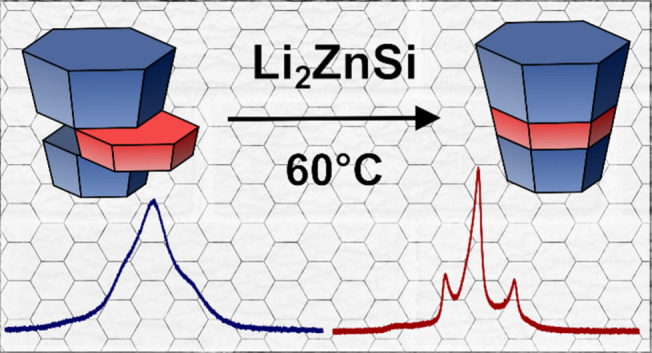

## 1 Introduction

Layered intermetallics and Zintl phases frequently exhibit weak interlayer interactions and highly directional bonding within the covalent slabs. As a result, many of these materials are sensitive to mechanical stress which can generate stacking faults, twins, or locally disturb cation positions. 2D disorder is documented in several classes of layered solids, including layered battery oxides,^1–3^ transition-metal dichalcogenides,^4,5^ graphite derived materials,^6,7^ and various Zintl phases,^8,9^ where relatively weak interlayer interactions allow local shear, gliding, or delamination under mechanical or electrochemical stress. Recent X-ray diffraction studies have shown that as-grown Li_2_ZnSi single crystals exhibit partial Zn occupancies, consistent with stacking faults or slab rotation of Zn–Si sheets.^9^ However, it remained unclear how the observed defect concentration relates to the synthesis temperature and the underlying thermodynamic conditions. If the defects were interpreted as quenched-in disorder originating from the configuration entropy at high temperatures, a concentration of about 4% would be unexpectedly large.^9^ Alternatively, the defect concentration might be influenced by mechanical stresses introduced after synthesis, a possibility that has not yet been examined in detail. In the present work, we demonstrate that the defect state of Li_2_ZnSi is rather mechanically induced during breaking, grinding, or pressing the samples into pellets. We show that stacking-fault disorder in Li_2_ZnSi heals out completely at surprisingly low temperatures of 310–370 K, far below typical annealing temperatures expected for intermetallic phases. Li_2_ZnSi thus represents a layered Zintl phase in which mechanically induced stacking faults can be removed by mild thermal treatment, providing a model system to study defect relaxation in mechanically sensitive 2D intermetallics.

## 2 Experimental Section

### 2.1 Preparation

Li_2_ZnSi was prepared following the procedure described in literature.^10,11^ Stoichiometric amounts of Li (99.95%), Zn (99.9999%), and Si (99.999%) were sealed in a Ta ampule under an Ar atmosphere. The mixture was heated at 770 °C (1043 K) for 7h and subsequently cooled to room temperature over 12 h. The reaction yielded air-sensitive, plate-like single crystals with a metallic luster. The same sample batch was already used as in a previous study, where phase purity was confirmed by powder X-ray diffraction (PXRD), scanning electron microscopy (SEM), and chemical analysis.^9^

### 2.2 Single-Crystal X-ray Diffraction (SCXRD)

A crystal sealed in an argon-filled quartz capillary was measured on a Rigaku Synergy S diffractometer with Mo–Kα radiation (λ = 0.71073 Å) and equipped with a hybrid pixel array detector (Dectris Eiger2 1M) at 293 and 373 K. Data integration and numerical absorption correction based on the ideal crystal structure description were performed using CrysAlisPro.^12^ The structure was solved by direct methods using SHELXS and refined with SHELXL.^13^ Crystallographic data have been deposited at the Fachinformationszentrum Karlsruhe, D-76344 Eggenstein-Leopoldshafen (Germany) under the depository numbers 2495697 and 2495747. Details regarding data collection and structure refinement (Tables S1–S3), atomic coordinates (Table S4), atomic displacement parameters (Table S5), and selected bond lengths (Table S6) are provided in the Supporting Information. The programs Atoms 6.3 and POV-Ray were used for structure drawings.

### 2.3 Heat Capacity

The substances were characterized using a double-furnace power-compensating differential scanning calorimeter (DSC 8500, PerkinElmer). Cold pressed pellets (*m* = 60 mg each) were placed in an Al_2_O_3_ crucible and covered with a lid. The measurements were performed over a temperature range of −40 °C K to 100 °C at a heating rate of 5 K/min under a helium flow of 100 mL/min. The He gas (99.999%) was further dried and purified from oxygen using an oxygen/moisture trap (Trigon Technologies). Temperature and heat flow were calibrated using an indium standard. The data were evaluated according to the sapphire method.

### 2.4 Nuclear Magnetic Resonance (NMR)

Experiments were performed on a Bruker Avance 500 spectrometer with a magnetic field of *B*_0_ = 11.74 T using standard Bruker static and magic-angle spinning (MAS) probes for 4 mm ZrO_2_ rotors. The ^7^Li and ^29^Si signals were referenced to LiCl and tetramethylsilane (TMS) with the reference frequencies of 194.366 and 99.361 MHz, respectively depending slightly on the probe used for the experiment. For the ^7^Li spectra, a single 90° pulse was used (duration of 1.6 μs; recovery time of 5 s). In case of ^29^Si, the Hahn-echo sequence (90°−τ–180°−τ– acquisition) was applied with 90° pulses of 6 μs and the recovery time of 1.6 s.

### 2.5 Impedance Measurements

The as-prepared Li_2_ZnSi was finely ground under an argon atmosphere using an agate mortar and subsequently compacted into a pellet by cold pressing (Ø = 10.0 mm; *d* = 2 mm). The measurements were performed using a PalmSens4 potentiostat. For electrochemical impedance spectroscopy (EIS), the pellet was positioned between two ion-blocking electrodes. An AC signal of 0.1 V was applied across a frequency range of 1.0 Hz to 100 kHz. Spectra were recorded between 298 and 323 K. Additionally, direct current (DC) polarization experiments were conducted at applied voltages of 50, 100, and 150 mV at room temperature.

### 2.6 Calculations

Electronic structure calculations were performed by using the all-electron, full-potential local orbital method, FPLO.^14^ The generalized gradient approximation to the density functional theory as parametrized according to ref 15. was employed. The structural optimizations were subjected to the force criterion of 5 meV Å^–1^. The equilibrium concentration of the defect layers was calculated using the canonical ensemble and the partition function approach (eq S1).

## 3 Results and Discussion

### 3.1 Defect State and Li Coordination: From Lonsdaleite to Diamond

The ideal crystal structure of Li_2_ZnSi (space group *P*6_3_/*mmc*) consists of planar Zn–Si layers arranged in an AB stacking sequence along [001] (Figure 1a). Lithium occupies interlayer 4*f* site above and below the centers of the Zn_3_Si_3_ rings and is additionally coordinated by a Si atom along [001]. The Li atoms form a nearly ideal tetrahedral network with lonsdaleite topology, even though neither the *c*/*a* ratio nor the free *z*-parameter of the Li 4*f* site enforces this geometry. In this topology, Li_6_ rings adopt boat conformations along [001] and chair conformations perpendicular to this direction, resulting in bicyclo[2.2.2]­octane-like cage motifs. A related lonsdaleite-type substructure with Na has been reported for the phase Na_2_MgSn.^16^ In the as-synthesized crystals, however, stacking faults disrupt the ideal AB stacking sequence of the Zn–Si layers, in which Zn atoms shift from the ideal 2*b* site into the center position 2*d* of the Zn_3_Si_3_ hexagons (Figure 2). This displacement is coupled to a migration of adjacent Li atoms from 4*f* to 4*e* positions, resulting in a distinct change in the local Li environment.^9^ In the ideal structure, each Li atom has a Si neighbor along [001], whereas in the defect configuration this axial neighbor is replaced by Zn (Figure S1). Thus, the defect introduces two chemically and electronically different Li coordination motifs. Moreover, the stacking fault induces a topological transformation of the Li sublattice. At the defect layer itself, the Li_6_-rings maintain the boat conformation along [001], as the Li atoms above and below move coherently with the displaced Zn atoms. In the next neighboring layers, however, all Li_6_ rings adopt chair conformations characteristic of the diamond topology (Figure 1b).

**Figure 1:**
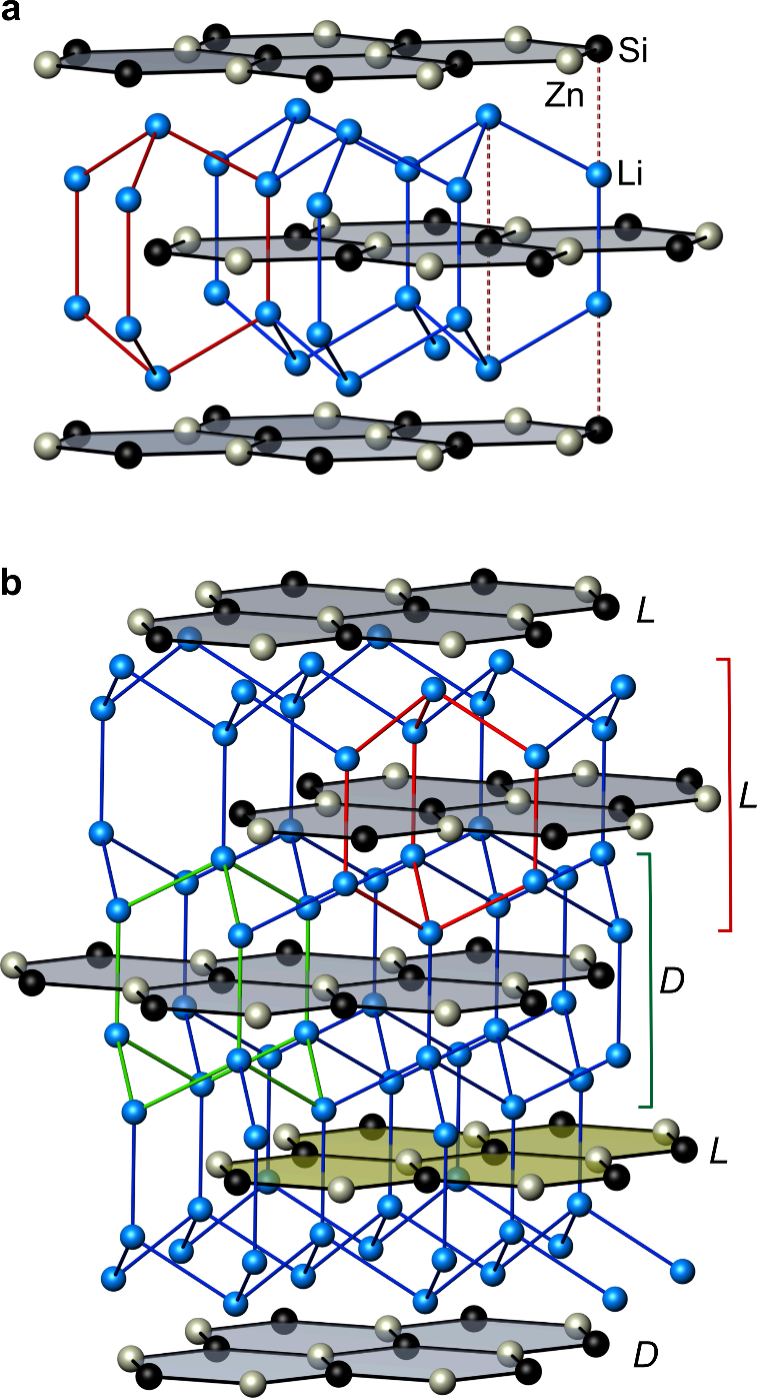
(a) Defect-free crystal structure of Li_2_ZnSi, featuring an interpenetrating assembly of a 3D lonsdaleite-type Li network intersected by Zn–Si layers. The characteristic bicyclo[2.2.2]­octane Li_6_ unit is highlighted in red. (b) Stacking fault in the Li_2_ZnSi structure: The combined shift of Zn atoms in a Zn–Si layer (highlighted in yellow) and adjacent Li atoms alters both the local Li coordination and the topology of the Li net. In Zn–Si layers neighboring the defect, the Li net adopts a diamond-type (*D*) arrangement instead of the lonsdaleite (*L*) structure. An adamantane cage is highlighted in green, and a bicyclo[2.2.2]­octane cage is highlighted in red.

**Figure 2:**
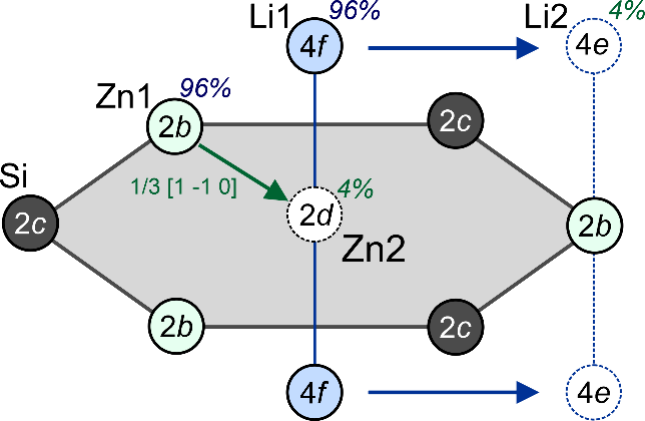
Scheme of the structural disorder of Li_2_ZnSi obtained from SCXRD experiments at room temperature^9^ caused by the shift of the Zn atoms (here: *z* = 3/4) and adjacent Li atoms.

This produces an alternation of lonsdaleite-like (L) and diamond-like (D) arrangement of Li atoms in the vicinity of the defect layer. Using the L/D notation, the local stacking sequence generated by a single fault ***L*** can be described as L–L–D–***L***–D–L–L, highlighting the spatial extent of the topology change. Therefore, a stacking fault of a Zn–Si layer creates multiple distinct Li environments such as L–L–L, L–L–D, or D–***L***–D, each associated with a different local bonding configuration and Li–Zn/Si coordination. In a crystal containing statistically distributed stacking faults, these environments lead to significant broadening of the ^7^Li NMR spectra, as observed experimentally (Figure 3). A hypothetical polymorph of Li_2_ZnSi in the rhombohedral space group *R*3*m* (Table S7), corresponding to an “all-defect” ABC stacking, would contain a fully diamond-like Li network, underscoring the intrinsic relationship between layer rotation and Li topology in Li_2_ZnSi (Figure S2).

**Figure 3:**
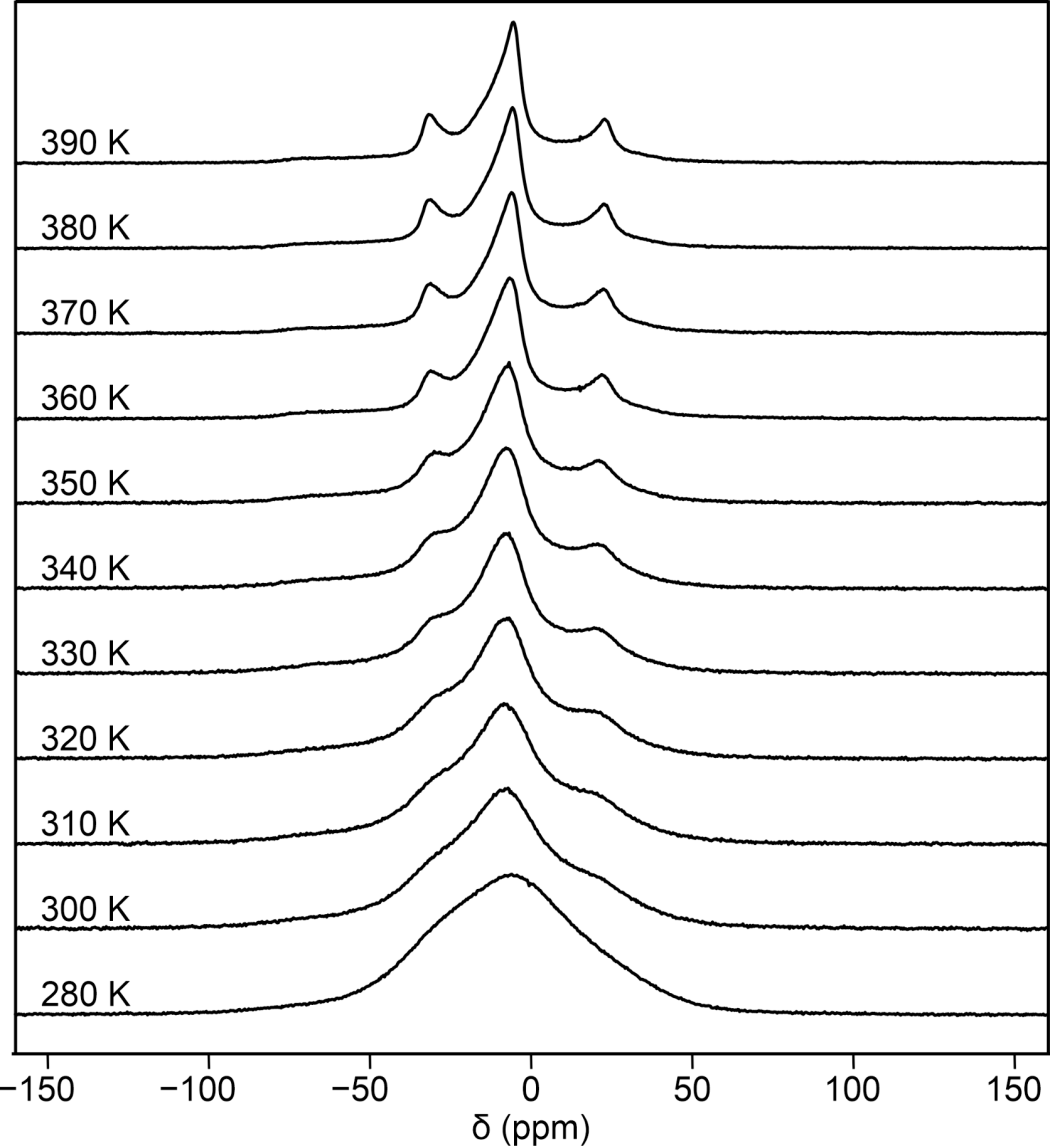
Static ^7^Li NMR spectra of Li_2_ZnSi. Upon heating, the initially broad resonance progressively narrows and develops into a well-resolved pattern, reflecting the temperature-activated healing of stacking-fault disorder.

After establishing the local defect model of Li_2_ZnSi, an important ambiguity remains. In a hexagonal lattice, the lateral shift of a Zn–Si layer is crystallographically equivalent to a 60° rotation of that layer, so that a stacking fault can alternatively be interpreted as the boundary of a rotational twin domain (Figure S3). Because both the split-site model and the two-domain rotational-twin model reproduce the room-temperature diffraction data equally well, SCXRD cannot uniquely resolve the spatial distribution of the faults.^9^ This limitation motivated the use of ^7^Li NMR as a sensitive probe of the distinct local Li environments created by such defects.

### 3.2 NMR Spectroscopy: Progressive Healing of Defects

#### 3.2.1 Temperature-Dependent ^7^Li NMR

The static ^7^Li NMR spectrum of freshly prepared Li_2_ZnSi at 280 K (Figure 3, bottom) exhibits a single, very broad resonance. Such extensive broadening is characteristic of a distribution of multiple local Li environments and indicates significant structural disorder at room temperature. Mechanical grinding prior to measurement further increases the line width, directly demonstrating the sensitivity of Li_2_ZnSi to mechanically induced defects. This observation is fully consistent with the structural model discussed above, in which stacking faults generate several distinct Li coordination environments. While room-temperature SCXRD cannot distinguish between a split-site model and a rotational twin model, ^7^Li NMR provides complementary information: The broad room-temperature line strongly suggests a locally disordered arrangement of stacking faults rather than a few well-ordered twin domains (Figure S3). Upon heating from 280 to 390 K, the broad featureless ^7^Li resonance narrows significantly and gradually develops distinct shoulders already at temperatures just above 300 K (Figure 3). This continuous evolution reflects a thermally activated relaxation of locally disordered Li environments. By 390 K, the spectrum resolves into three distinct peaks, consistent with a single crystallographic Li site with well-defined local symmetry. At intermediate temperatures, healed and defective regions coexist. As shown by the HT-SCXRD results discussed below, the stacking-fault disorder is fully removed at the temperatures at which the ^7^Li NMR spectra resolve.

#### 3.2.2 ^7^Li NMR Spectrum at 390 K

At 390 K, the ^7^Li NMR spectrum resolves into three distinct peaks, whose line shape is consistent with a single crystallographic Li site in an ordered phase with well-defined local symmetry. In the ideal crystal structure of Li_2_ZnSi, the ^7^Li nuclei (*I* = 3/2) occupy the 4*f* position with axial symmetry (3*m.*). This requires consideration of both quadrupole coupling and magnetic shift anisotropy. The quadrupole interaction arises from the coupling between the nuclear quadrupole moment (*Q*) and the electric field gradient (*eq*) at the Li site, quantified by the coupling constant *C*_q_ = *e* ^2^ *Qq*/h. The quadrupole interaction splits the ^7^Li signal into three distinct contributions: The central transition (*m* = +1/2 ↔ −1/2), which is unaffected by first-order quadrupole effects, and two satellite transitions (*m* = ±3/2 ↔ ±1/2), flanking the central line (Figure 4).

**Figure 4:**
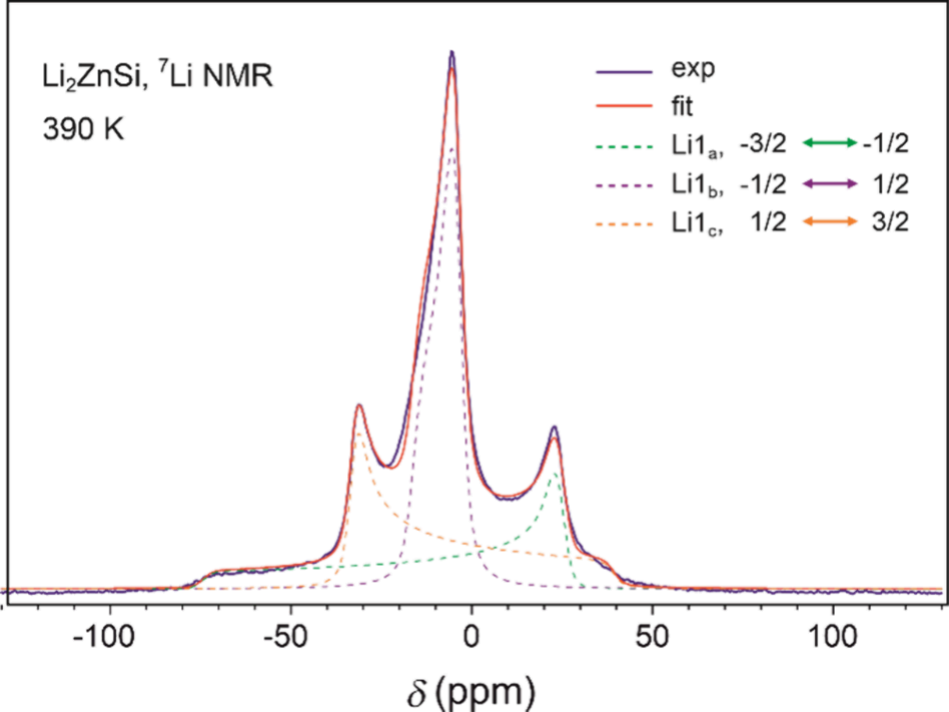
Static ^7^Li NMR spectrum of Li_2_ZnSi without stacking faults at 390 K.

The first-order quadrupole frequency shift for spin transitions *m* → *m* – 1 in a given crystallite is Δν_q_(*m*) = −(e^2^ *Qq*/2h)­(*m* – 1/2)­(3cos^2^θ – 1 + η sin^2^θcos2ϕ), where η is an asymmetry parameter, while θ and ϕ describe the orientation of the applied magnetic field with respect to the principal-axes system (PAS) of the electric field gradient tensor. Due to the axial symmetry of ^7^Li nuclei in Li_2_ZnSi, the latter equation is reduced to Δν_q_(*m*) = −(*e* ^2^ *Qq*/2*h*)­(*m* – 1/2)­(3cos^2^θ – 1), where θ is the angle between the [001] axis of the crystallite and the external magnetic field. We consider the quadrupole shift in relative terms as δ_quad,*m*_ = Δν_q_(*m*)/ν_L_ × 10,^6^ where ν_L_ is the resonance frequency of a reference compound, e.g., of Li^+^ in aqueous solution. Given the small value of *C*_q_, second-order quadrupole perturbations to the central transition are negligible in this case. In addition to quadrupole splitting, the resonance frequencies of all three transitions are influenced by local magnetic field perturbations arising from the chemical environment of the Li nucleus (chemical shift, δ_chem_), and hyperfine coupling between the nuclear spins and the conduction electrons (Knight shift in metallic systems, *K*). These two effects induce a magnetic frequency shift (Δν_mag_), which scales linearly with the external magnetic field (*B*_0_). It can be expressed in units of ppm as δ_mag_ = δ_chem_ + *K* = Δν_mag_/ν_L_ × 10,^6^ a ratio of the total frequency shift and ν_L_. The chemical shift and the Knight shift depend on the orientation of the crystallites relative to the external magnetic field, which is for the chemical shift expressed as δ_chem_ = δ_iso_ + δ_aniso_/2­(3cos^2^θ′ – 1 + ε sin^2^θ′cos2ϕ′)). Here, δ_iso_ is the isotropic chemical shift, while the chemical shift anisotropy (CSA) is determined by δ_aniso_ and ε, which are the largest principal value and the asymmetry parameter of the traceless anisotropic chemical shift tensor. The angles θ′ and ϕ′ express the orientation of the magnetic field with respect to the PAS of the chemical shift tensor. The Knight shift is described by a similar equation with a set of parameters *K*_iso_, *K*_aniso_, ε′ and its own PAS. Due to the axial symmetry of ^7^Li nuclei in Li_2_ZnSi the parameters ε and ε′ are equal to 0 and the orientations of the PAS for both chemical and Knight shift are aligned along the [001] axis of the crystallites. This results in the total magnetic shift of δ_mag_ = (δ_iso_ + *K*_iso_) + (δ_aniso_ + *K*_aniso_)/2­(3cos^2^θ′ – 1). The total relative frequency shift for a given quadrupole transition is therefore δ_total,*m*_ = δ_quad_ + δ_mag_. To obtain the complete spectrum, the summation of all three quadrupole transitions (weighted by a factor β_*m*_ = *I*(*I* + 1) – *m*(*m* – 1)) and all spatial orientations of the crystallites is performed. Additional broadening of the spectral line shape arises from field inhomogeneity (instrumental broadening), dipolar coupling between nuclear spins, and local environment variations of the Li atoms caused by structural defects. To account for these effects, we employ a pseudo-Voigt function PV­(*x*) = η *L*(*x*) + (1 – η) *G*(*x*) for spectral simulation, where *L*(*x*) is the Lorentzian component (homogeneous broadening), *G*(*x*) the Gaussian component (inhomogeneous broadening), and η the mixing parameter.

The 390 K spectrum was successfully modeled by incorporating first-order quadrupole coupling (*C*_q_ = 22 kHz), isotropic (δ_iso_ = – 8 ppm) and anisotropic (δ_aniso_ = – 9 ppm) magnetic shifts, and pseudo-Voigt broadening (Δδ = 4.7 ppm). Δδ represents the intrinsic line width, independent of quadrupolar and anisotropic magnetic contributions. The values of this isotropic chemical shift and the isotropic Knight shift cannot be determined individually. However, the Knight shift is always a positive and relatively large contribution, reaching approximately 250 ppm in Li metal. Despite the metallic character of Li_2_ZnSi,^9^ the 390 K spectrum shows a negative isotropic magnetic shift. Therefore, the contribution of the Knight shift to the magnetic shift is negligible, which indicates that charge transfer from Li to the Zn–Si layers is essentially complete.^17^ For the analysis of the spectra between 280–380 K, *C*_q_ and δ_aniso_ were held constant at the values determined from the 390 K fit, while δ_iso_ and Δδ were allowed to vary. This approach enabled quantitative extraction of the temperature-dependent line broadening (Figure S4).

#### 3.2.3 ^29^Si NMR Spectrum

Due to the low natural abundance and NMR sensitivity of the ^29^Si isotope, only a room-temperature NMR spectrum was recorded (Figure 5). The magic angle spinning (MAS) spectrum displays a single, broad resonance at 155 ppm, indicative of a significant Knight shift and confirming the metallic character of Li_2_ZnSi.^17^ The static spectrum exhibits a broader, asymmetric line-shape with a maximum around 120 ppm. Ideally, a CSA-broadened powder pattern for an I=12 nucleus like ^29^Si on the axially symmetric site would exhibit a maximum at one edge of the spectral line and a minimum at the other, corresponding to the 90° and 0° orientations of the *z*-axis of the crystallites relative to the external magnetic field. The observed deviation from the ideal CSA line-shape and the considerable line width of the MAS signal strongly suggests the presence of structural disorder as the dominant source of broadening. Thus, both ^7^Li and ^29^Si NMR independently indicate pronounced local structural disorder in the as-prepared material.

**Figure 5:**
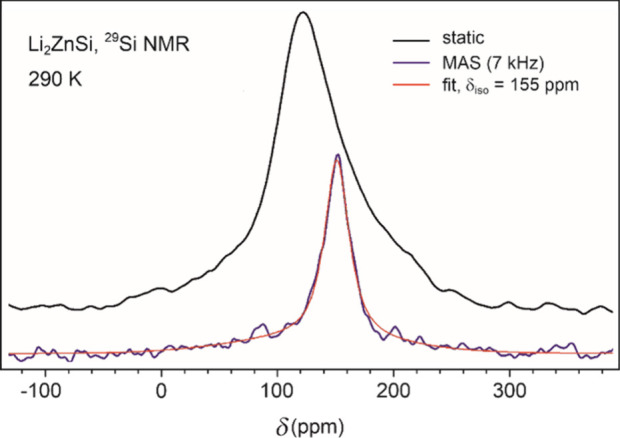
Static and magic angle spinning ^29^Si NMR spectra of Li_2_ZnSi.

### 3.3 Structural Response to Mild Heating: High-Temperature X-ray Diffraction

The structure of Li_2_ZnSi consists of planar Zn–Si layers separated by Li atoms and crystallizes in the hexagonal space group *P*6_3_/*mmc* (*Z* = 2; *a* = 4.2458(2) Å, *c* = 8.224(1) Å). In the ideal configuration, Zn atoms occupy Wyckoff position 2*b* and the Si atoms 2*c*. The Li atoms at position 4*f* are located above and below the center of each Zn_3_Si_3_ ring. A previous investigation^9^ did not reveal evidence of the previously proposed supercell, symmetry reduction, or layer puckering.^10^ However, single crystals investigated at room temperature consistently revealed structural disorder.^9^ For the highest-quality crystal (*R*(*F* ^2^) = 0.04), refinements showed only 96% occupancy of Zn at its expected site (Zn1 at 2*b*), while 4% of the Zn atoms are displaced to the centers of the Zn_3_Si_3_ rings (Zn2 at 2*d*). However, this displacement is sterically incompatible with Li on 4*f* and forces Li into the adjacent 4*e* site, yielding a local rearrangement of the Li sublattice. To determine whether this defect state is intrinsic or can be removed by heating, a single crystal from the disordered batch was examined by X-ray diffraction at 100 °C (≈373 K). The temperature was chosen based on the temperature-dependent ^7^Li NMR spectra (Figure 3).

At this temperature, refinement yields an excellent model with fully ordered Zn and Li positions (*R*(*F* ^2^) = 0.017; Table S2). Both Zn and Li split positions disappear entirely (Table S4), demonstrating that the stacking faults seen at room temperature are not thermodynamically stable but anneal out upon moderate heating. Subsequent cooling preserves the ordered structure, although the refinement residuals slightly increase (*R*(*F* ^2^) = 0.025; Table S3), demonstrating permanent recovery of the ordered structure on the experimental time scale. The small increase may arise from slight kinetic trapping of residual disorder or weak interaction with the capillary environment. The thermal expansion of Li_2_ZnSi between 300 and 370 K is small (Δ*a* = 0.02%, Δ*c* = 0.03%) (Table S1). These results demonstrate that the defects observed in the crystals before heating are mechanically induced and are fully removed by mild thermal treatment.

### 3.4 Heat Capacity: A Broad Relaxation Process

The specific heat capacity of Li_2_ZnSi revealed a weak endothermic anomaly upon heating, starting at approximately 50 °C and reaching a broad maximum near 70 °C (Figure 6, top). The signal has the characteristic shape of a “relaxation hump” indicating that the process is not associated with a structural phase transition but instead reflects a continuous, thermally activated relaxation. No anomaly is observed upon cooling, and the measurement is reproducible for two independent samples. The absence of a cooling peak might suggest that the underlying mechanism involves the annealing of configurational disorder, consistent with the temperature-dependent NMR and HT-SCXRD data. However, when the heat-capacity measurement is repeated after cooling the sample (without removing it from the instrument) an essentially identical endothermic signal is observed again (Figure 6, bottom). The thermal anomaly observed upon heating cannot originate from the ordering of stacking faults, as these defects are already removed during the first heating cycle, as shown by the SCXRD experiments. Instead, the anomaly is interpreted as reflecting the onset of ionic mobility, while the removal of stacking-fault disorder is regarded as a secondary consequence of this process. The relatively low temperature of the anomaly (≈70 °C) is consistent with reported activation energies for Li-ion conductors.^18^ This model might also explain the slightly higher R1-value of the cooled single-crystal XRD data set. As the mobility decreases upon cooling, a fraction of the atoms becomes kinetically trapped in suboptimal positions, generating a small number of frozen-in defects. The absence of a cooling anomaly can be explained with noncooperative freezing of the mobile atomic species. Upon cooling, the ionic mobility decreases gradually, such that individual ions relax into local energy minima over a broad temperature range, preventing the appearance of a distinct calorimetric feature.

**Figure 6:**
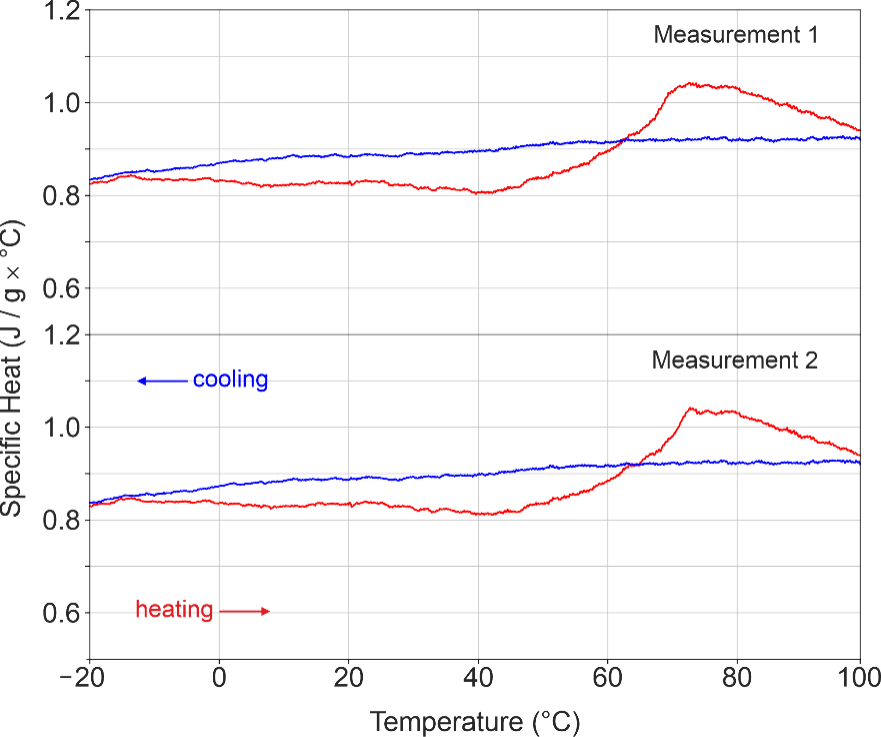
Heat-capacity curves of Li_2_ZnSi. Both heating cycles show the same broad endothermic anomaly, while no feature appears on cooling.

### 3.5 Quantum Mechanical Investigations

#### 3.5.1 Stability of the Planar Zn–Si Layers

For the crystal structure of Li_2_ZnSi, different models and space groups have been proposed in the literature. In addition to the model in the space group *P*6_3_/*mmc* (No. 194) with planar Zn–Si layers, which is verified in this work, a model with corrugated Zn–Si layers was suggested earlier.^10^ Since distortions and symmetry reductions may occur at low temperatures, a quantum-mechanical optimization of the Li_2_ZnSi structure was performed. This optimization can be considered an approximation of the 0 K state, since these first-principles calculations involve only electronic total energies and do not account for entropic contributions. The optimization was carried out in subgroup *P*3*m*1 (No. 156), which, in contrast to *P*6_3_/*mmc*, allows for puckering because all Wyckoff positions entail a free *z*-parameter. The unit cell volume was kept constant, but the *c/a* ratio was optimized. The optimized structure retains perfectly planar Zn–Si layers (Δ*z* < 2 × 10^–5^) and exhibits only minor Li displacements relative to experiment (Table S7). The optimum *c*/*a* ratio being 1.9322 agrees closely with the experimental value of 1.937. These results indicate that a distortion of the Zn–Si layers is not energetically favored and the P6_3_/*mmc* structure model remains valid for low temperatures as well. Thus, the low-temperature heat-capacity anomaly is consistent with defect relaxation rather than a symmetry change by puckering of the Zn–Si layers.

#### 3.5.2 Localization of Stacking-Fault Defects

Experimentally, room-temperature Li_2_ZnSi crystals exhibit stacking faults. On average, one in every 25 Zn–Si layers is displaced (1/25 = 4%). To study the energetic impact of such defects, we investigated 1 × 1 x *N*_*sc*_ supercells (2 < *N*_*sc*_ < 9) in space group *P3m1* (no. 156), each constructed with a single displaced Zn–Si layer. Considering that there are two Zn–Si layers in the ideal unit cell, the investigated largest super cell, *N*_*sc*_ = 9, corresponds to a defect concentration of 1/18 ≈ 5.6%, which is close to the experimental result.^9^ The defect formation energy (Δ*E*_def_) was calculated as the difference between the total energies of the super cell containing one defect layer and the ideal structure. Remarkably, Δ*E*_def_ is essentially independent of the supercell size, and hence of the defect concentration (Figure 7). The average of the computed values is Δ*E*_def_ = 0.405 ± 0.002 eV. This independence implies that electronically the layer defect is highly localized. Consistent with this picture, the electronic DOS, computed exemplary for *N*_sc_ = 6 and 9 indicate that the DOS of the atoms neighboring the defect layer are more similar to the DOS of the atoms in the more distant layers (Figure S5). The computed short-range energetic impact of the defects also explains why such defects can be annealed at unexpectedly low temperatures: only the displaced layer must relax, without cooperative motion of the stack.

**Figure 7:**
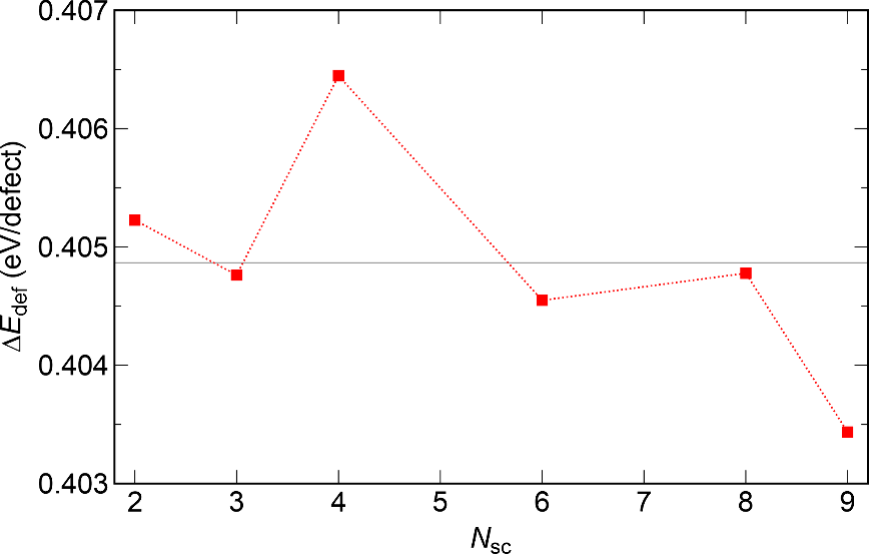
Calculated defect formation energy (Δ*E*_def_) as a function of supercell size (1 × 1 × *N*_sc_). Increasing *N*_sc_ corresponds to decreasing defect concentration. The determined average defect formation energy is Δ*E*_def_ ≈ 0.405 eV.

The concentration independence of Δ*E*_def_ suggests additivity of defect energies, i.e., the total energy increases linearly with the number of defect layers. This idea was checked by calculations performed on 1 × 1 × 6 super cells having two defect layers separated by an interlayer distance of *d*. Note that, two neighboring (Zn, Si) layers, *d* = *c/*2 apart, cannot contain defects simultaneously, because otherwise the associated migration of the Li atoms would result in a very short Li–Li distance of 1.36 Å. Hence, calculations were carried out for *d* = *c*, 3*c*/2, 2*c,* and 5*c*/2. The maximum deviation of the double defect formation energy from twice the single defect formation energy (0.405 eV) is less than 6%, substantiating the linear scaling of the total energy with the number of defects.

#### 3.5.3 Equilibrium Defect Concentrations

Using the defect formation energy and a configurational-entropy model (SI), the equilibrium defect concentration *c*_eq_(*T*) was estimated. At the highest reaction temperature of 770 °C (1043 K), the calculated defect concentration is only about 1% and thus significantly lower than the experimental value of 4% observed at room-temperature. Although the calculated defect concentration may be underestimated due to neglected entropy contributions (e.g., phonons), the deviation is fully consistent with the model that the experimentally observed defects predominantly arise from mechanical manipulation after synthesis, which readily introduces stacking faults. In any case, the observed defect concentration at room temperature represents a kinetically frozen state, not the thermal equilibrium concentration. At 370 K, the crystals are still practically free of defects (*c*_eq_(*T*) = 0.0003%) which is confirmed in the high-temperature X-ray diffraction experiments.

### 3.6 Impedance Spectroscopy: Grain-Boundary-Dominated Transport

Previous investigations of single-crystalline Li_2_ZnSi have demonstrated metallic behavior with a positive temperature coefficient of resistivity (dρ/d*T* > 0) and high electrical conductivity (σ­(300 K) = 8.5 × 10^3^ S/cm), consistent with the calculated electronic density of states.^9,17^ However, polycrystalline pellets obtained by milling and cold-pressing material from the same batch show semiconducting behavior and several orders of magnitude lower conductivity (1.7 × 10^–7^ S/cm). This discrepancy is typical for mechanically sensitive intermetallic phases, where grain boundaries, microcracks, and surface oxidation strongly suppress electronic transport. Impedance spectra of the pressed pellets (298–323 K) display a single, slightly depressed semicircle in Nyquist representation (Figure 8). Such a response is characteristic of grain-boundary-dominated conduction, rather than intrinsic bulk transport. The relaxation reflects the effective response of a microstructurally disordered network of grains, not the intrinsic metallic properties of Li_2_ZnSi. The ionic conductivity extracted at 300 K (≈3 × 10^–7^ S/cm) and the activation energy derived from the Arrhenius fit (≈ 0.77 eV) should therefore be interpreted as effective parameters, representing interfacial resistance, limited percolation pathways, and possible surface oxidation. However, the measurements reinforce the central conclusion that Li_2_ZnSi is extremely sensitive to mechanical treatment: grinding and pressing disrupt the electronically conductive pathways and create a microstructure in which grain boundaries dominate the AC response. The impedance data therefore complement the NMR and X-ray diffraction results by demonstrating that mechanical manipulation not only induces stacking faults but also profoundly alters the electrical transport behavior.

**Figure 8:**
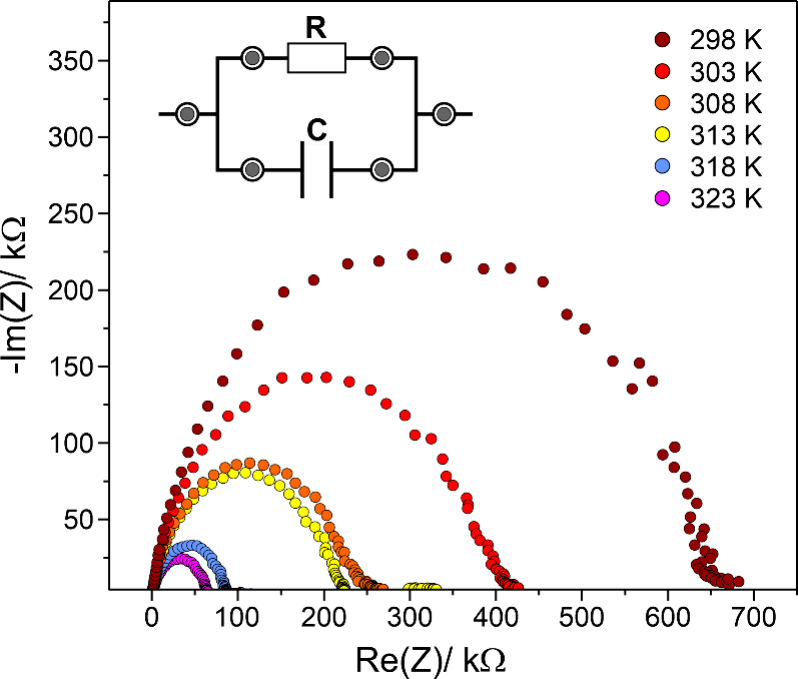
Impedance spectra of a polycrystalline Li_2_ZnSi sample after milling and pressing. The spectra, displayed in Nyquist representation, were recorded between 298 and 323 K.

## 4 Conclusions

Li_2_ZnSi represents a layered 2D compound in which mechanically induced stacking faults can be fully healed at exceptionally low temperatures. The defects are highly localized within individual Zn–Si layers and have a low formation enthalpy. Permanent recovery of the ordered P6_3_/*mmc* structure is demonstrated exclusively by high-temperature and subsequent cooling single-crystal X-ray diffraction, while ^7^Li NMR sensitively probes the relaxation of locally disordered Li environments. Stacking faults in as-synthesized crystals represent a kinetically frozen metastable state, while the defect-free structure is thermodynamically stable at room temperature. Grinding and pressing not only introduce stacking faults but also disrupt the electronically conductive pathways and mask the intrinsic metallic behavior.

## Supplementary Material



## Notes

S1: Lattice parameters of the Li_2_ZnSi specimen; S2: crystallographic data for Li_2_ZnSi (373 K); S3: crystallographic data for Li_2_ZnSi (293 K); S4: atomic coordinates; S5: anisotropic displacement parameters; S6: selected bond lengths; S7: calculated concentration of stacking faults; S8: local Li coordination environment; S9: crystallographic data of a hypothetical polytype of Li_2_ZnSi in the space group *R*3*m*; S10: structure model in the space group *R*3*m*; S11: alternative models for stacking faults; S12: line widths of temperature-dependent ^7^Li NMR spectra; S13: optimized structure model in the space group *P*3*m*1; and S14: atom-projected DOS computed for the 1 × 1 × 6 and 1 × 1 × 9 supercells (PDF)
