# Supplementary material for: Low-Temperature Defect Healing in the Layered Zintl Phase Li2ZnSi
Source: Inorg Chem. 2026 Jan 27;65(5):3147–54. doi: 10.1021/acs.inorgchem.5c05692 (PMC12892307; doi:10.1021/acs.inorgchem.5c05692)
Supplement: Supplementary file 1 [file ic5c05692_si_001.pdf]

Supporting Information

Low-Temperature Defect Healing in the Layered Zintl Phase Li_2ZnSi

Xian-Juan Feng[1], Matej Bobnar[2], Alim Ormeci[3], Mohammad Mehmandoust[4], Marcus Schmidt[3], Bodo Böhme[3], Mitja Krnel[3], Michael Baitinger[3], Julia Maria Hübner[4*]

[1]Institute of Nonferrous Metallurgy and Purest Materials

TU Bergakademie Freiberg

Leipziger Straße 34, 09599 Freiberg, Germany

[2]Krka, d. d.

Šmarješka cesta 6,

8000 Novo mesto, Slovenija

[3]Max Planck Institute for Chemical Physics of Solids

Nöthnitzer Strasse 40

01187 Dresden, Germany

[4]Technische Universität Dresden

Fakultät für Chemie und Lebensmittelchemie

Bergstraße 66

01069 Dresden, Germany

Email: Julia-Maria.Huebner@tu-dresden.de

Supporting Information

Table of content:

**Table S1**<br>Lattice Parameters of Li_2ZnSi specimen	S2
**Table S2**<br>Crystallographic data for Li_2ZnSi (373(2) K)	S3
**Table S3**<br>Crystallographic data for Li_2ZnSi (293(2) K; "RT-II")	S4
**Table S4**<br>Atomic coordinates	S5
**Table S5**<br>Anisotropic displacement parameters	S6
**Table S6**<br>Selected bond lengths in Li_2ZnSi	S7
**Equations S1**<br>Concentration of stacking faults	S8
**Figure S1**<br>Local Li coordination environment before and after the Zn-Si layer shift	S10
**Table S7**<br>Crystallographic data of Li_2ZnSi in space group *R*3*m*	S10
**Figure S2**<br>Structure model of Li_2ZnSi in space group *R*3*m*	S11
**Figure S3**<br>Alternative models for stacking faults	S12
**Figure S4**<br>Linewidths of Temperature-dependent ^7Li NMR spectra	S12
**Table S7**<br>Optimized structure model for Li_2ZnSi in space group *P*3*m*1	S13
**Figure S5**<br>Atom-projected DOS computed for the 1 x 1 x 6 and 1 x 1 x 9 supercells	S14

Three crystal structure determinations of Li_2ZnSi are discussed in this work:

- Crystals obtained from a sample cooled within 12 h from 770 °C to room temperature were measured at **293 K** (Sample "**RT-I**"). These results were previously reported and are used here as a reference for comparison.

- A crystal from the same batch was measured at **373 K**.

- The same crystal was measured again after cooling to **293 K** (Sample "**RT-II**")

Table S1. Lattice Parameters of Li_2ZnSi specimen.

Sample	Lattice parameter	Method
Li_2ZnSi (295 K, RT-I)	*a* = 4.2458(2) Å; *c* = 8.224(1) Å	PXRD, LaB_6 standard
Li_2ZnSi (373°K)	*a* = 4.25145(7) Å; *c* = 8.2444(2) Å	single crystal
Li_2ZnSi (295 K, RT-II)	*a* = 4.2452(1) Å; *c* = 8.2258(2) Å	single crystal

Table S2. Crystallographic data for Li_2ZnSi (373(2) K).

Composition	Li_2ZnSi
Molar mass / g mol^{-1}	107.34
Crystal system; space group	Hexagonal, $P6_3/mmc$ (no. 194)
a / Å	4.2515(1)
c / Å	8.2444(2)
V / Å^3	129.05(1)
Z; ρ_{calc} / (g cm^{-3})	2; 2.762
Diffractometer	Rigaku AFC7, Saturn 724+CCD detector
λ / Å	0.71073 (Mo$K\alpha$)
Temperature / K	373(2)
Crystal shape	blocky fragment
Crystal size / mm	0.36 × 0.28 × 0.25
μ / mm^{-1}	9.569
Extinction coefficient	0.28(3)
θ range / deg.	4.945 - 35.388
Indexes ranges	$-6 \le h \le 6$, $-6 \le k \le 6$, $-13 \le l \le 13$
F(000) / e	100
Reflection measured, independent	5867; 137 [R_{int} = 0.0576]
Data / restraints / parameters	137 / 0 / 9
Goodness-of-fit on F^2	1.412
Final R indices [$I > 2\sigma(I)$]	$R1$ = 0.0177, w$R2$ = 0.0534
R indices (all data)	$R1$ = 0.0179, w$R2$ = 0.0535
Largest diff. peak and hole	0.578 and –0.470 e/Å^3

Table S3. Crystallographic data for Li_2ZnSi (293(2) K; “**RT II**“).

Composition	Li_2ZnSi
Molar mass / g mol^{-1}	107.34
Crystal system; space group	Hexagonal, $P6_3/mmc$ (no. 194)
a / Å	4.2453(1)
c / Å	8.2258(2)
V / Å^3	128.39(1)
Z; ρ_{calc} / (g cm^{-3})	2; 2.777
Diffractometer	Rigaku AFC7, Saturn 724+CCD detector
λ / Å	0.71073 (Mo$K\alpha$)
Temperature / K	293(2)
Crystal shape	blocky fragment
Crystal size / mm	0.36 × 0.28 × 0.25
μ / mm^{-1}	9.619
Extinction coefficient	0.47(5)
θ range / deg.	4.957 - 33.611
Indexes ranges	$-6 \le h \le 6$, $-6 \le k \le 6$, $-13 \le l \le 13$
F(000) / e	100
Reflection measured, independent	3173; 123 [R_{int} = 0.0711]
Data / restraints / parameters	137 / 0 / 9
Goodness-of-fit on F^2	1.157
Final R indices [$I > 2\sigma(I)$]	$R1$ = 0.0243, w$R2$ = 0.0569
R indices (all data)	$R1$ = 0.0244, w$R2$ = 0.0570
Largest diff. peak and hole	0.645 and –0.745 e/Å^3

Table S4. Atomic parameters of Li_2ZnSi measured at 293 K (RT-I and RT-II) and 373 K.

**Sample**	**Zn1** (2*b*)	**Zn2** (2*d*)	**Li1** (4*f*)	**Li2** (4*e*)	**Si** (2*c*)
	0 0 1/4	1/3 2/3 3/4	1/3 2/3 z_1	0 0 z_2	1/3 2/3 1/4
Li_2ZnSi (RT-I)	0.963(5)	0.04(4)	0.962(5)<br>z_1 = 0.919(1)	0.04(4)<br>z_2 = z_1	1
Li_2ZnSi (393 K)	1	-	1<br>z_1 = 0.9176(7)	-	1
Li_2ZnSi (RT-II)	1	0*	1<br>z_1 = 0.9173(9)	-	1

*Occupancy was refined to 0.0012(4)

Table S5. Anisotropic displacement parameters ($Å^2$ x 10^3) for Li_2ZnSi. The anisotropic displacement factor exponent takes the form: $-2\pi^2[h^2 a^{*2}U_{11} + ... + 2 h k a^* b^* U_{12}]$.

a) Dataset collected at 295 K (RT-I).

**Atom**	U_{11}	U_{22}	U_{33}	U_{23}	U_{13}	U_{12}
Zn1	15(1)	U_{11}	44(1)	0	U_{23}	7(1)
Si1	14(1)	U_{11}	31(1)	0	U_{23}	7(1)
Li1	19(3)	U_{11}	32(5)	0	U_{23}	10(2)

b) Dataset collected at 373 K.

**Atom**	U_{11}	U_{22}	U_{33}	U_{23}	U_{13}	U_{12}
Zn1	10(1)	U_{11}	28(1)	0	U_{23}	5(1)
Si1	8(1)	U_{11}	11(1)	0	U_{23}	4(1)
Li1	23(2)	U_{11}	18(2)	0	U_{23}	12(1)

c) Dataset collected at 295 K (RT-II) after cooling from 373 K.

**Atom**	U_{11}	U_{22}	U_{33}	U_{23}	U_{13}	U_{12}
Zn1	10(1)	U_{11}	25(1)	0	U_{23}	5(1)
Si1	9(1)	U_{11}	11(1)	0	U_{23}	5(1)
Li1	25(3)	U_{11}	12(3)	0	U_{23}	13(1)

Table S6. Selected bond lengths (*d*/Å) in Li_2ZnSi from structure refinement of datasets collected at 293 K (RT-II) and 373 K. The distances obtained from the datasets RT-I and RT-II do not differ within experimental error.

Interatomic distances (d/Å)		373 K	293 K
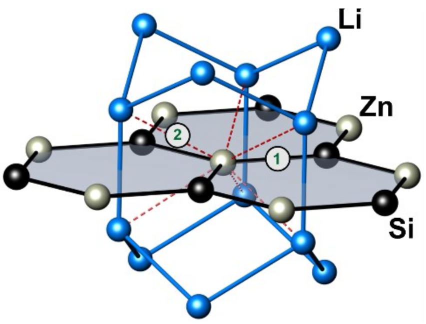	① Zn – Si 3x	2.4546(1)	2.4510(1)
	② Zn – Li 6x	2.817(3)	2.811(4)
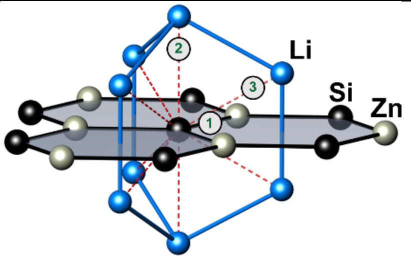	① Si – Zn 3x	2.4546(1)	2.4510(1)
	② Si – Li 2x	2.740(6)	2.736(7)
	③ Si – Li 6x	2.817(3)	2.811(4)
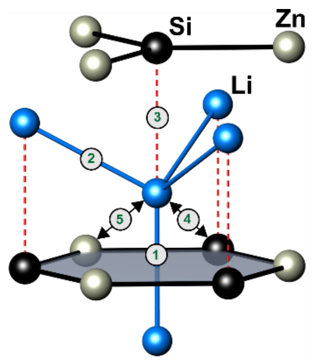	① Li – Li 1x	2.76(1)	2.75(2)
	② Li – Li 3x	2.805(6)	2.803(7)
	③ Li – Si 1x	2.740(6)	2.736(7)
	④ Li – Si 3x	2.817(3)	2.811(4)
	⑤ Li – Zn 3x	2.817(3)	2.811(4)

Equation S1. Concentration of stacking faults

The equilibrium concentration of defects is determined via statistical mechanics utilizing the canonical ensemble and its corresponding partition function. For the case of planar defects in Li_2ZnSi, we analyze a unit cell comprising $M = 2\ N_{supercell}$ layers. The defect layers (Zn, Si) are subject to a crystallographic constraint: they cannot be successive because such an arrangement would result in an unphysically short distance (1.36 Å) between co-displaced Li atoms. Therefore, for l defect layers, the number of configurations having successive defect layers should be subtracted from the unrestricted defect distribution number, $M!\ /\ [l!\ \cdot\ (M\text{-}l)!]$. Another consequence of the crystallographic restriction is that the that maximum allowed value of l is $M/2$. We have

$$Z = \sum_{l=0}^{M/2} g_l\, e^{-E(l)/k_B T}$$

with the multiplicity factor g_l and the exponential term is the Boltzmann factor. $E(l)$ is the energy of the structure with l defect layers. It can be expressed as $E_{\text{def}}\,(l) = E(l) - E_{\text{ideal}}$. Since the defect formation energy is independent of defect concentration, $E_{\text{def}}\,(l) = l \cdot E_{\text{def}}$ can be assumed. Then, $E(l) = E_{\text{def}}\,(l) + E_{\text{ideal}} = l \cdot E_{\text{def}} + E_{\text{ideal}}$, however the energy of the pristine (ideal) structure is a constant and it does not affect the thermal average values. We get

$$Z = \sum_{l=0}^{M/2} g_l\, e^{-\,l\,E_{def}/k_B T}$$

The average value of defect layers at a temperature T, $<l>_T$, is given by

$$< l >_T = Z^{-1} \cdot \sum_{l=0}^{M/2} l\, g_l\, e^{-\,l\,E_{def}/k_B T} = Z^{-1} \cdot \sum_{l=1}^{M/2} l\, g_l\, e^{-\,l\,E_{def}/k_B T}$$

The multiplicity factors for 0 and 1 defect layer case are g_0 = 1 and g_1 = *M*. When *l* = 2, *M* pairs of successive defect layers are possible, and they should be excluded. Thus,

$$g_2 = M! / [2! \cdot (M\text{-}2)!] - M = M \cdot (M\text{-}3) / 2$$

Reasoning in a similar way for higher numbers of defect layers, we find the following expression for the general case:

$$g_l = \frac{M}{l!} \cdot \frac{(M-l-1)!}{(M-2l)!}$$

The full expression for the partition function becomes

$$Z = \sum_{l=0}^{M/2} \frac{M}{l!} \cdot \frac{(M-l-1)!}{(M-2l)!}\, e^{-\,l\,E_{def}/k_B T}$$

The average number of defect layers at *T* is obtained as

$$< l >_T = Z^{-1} \cdot \sum_{l=1}^{M/2} l\,\frac{M}{l!} \cdot \frac{(M-l-1)!}{(M-2l)!}\, e^{-\,l\,E_{def}/k_B T}$$

$$< l >_T = \frac{M}{Z} \cdot \sum_{l=1}^{M/2} \frac{1}{(l-1)!} \cdot \frac{(M-l-1)!}{(M-2l)!}\, e^{-\,l\,E_{def}/k_B T}$$

The equilibrium concentration of defect layers at a temperature *T* is $c_{eq}(T) = <l>_T / M$. Hence, this gives us the desired expression:

$$c_{eq}(T) = \frac{1}{Z} \cdot \sum_{l=1}^{M/2} \frac{1}{(l-1)!} \cdot \frac{(M-l-1)!}{(M-2l)!}\, e^{-\,l\,E_{def}/k_B T}$$

Z and $c_{eq}(T)$ are evaluated numerically. The exponential term gets very small with increasing *l*, enabling converged results even for *l* ≤ 5.

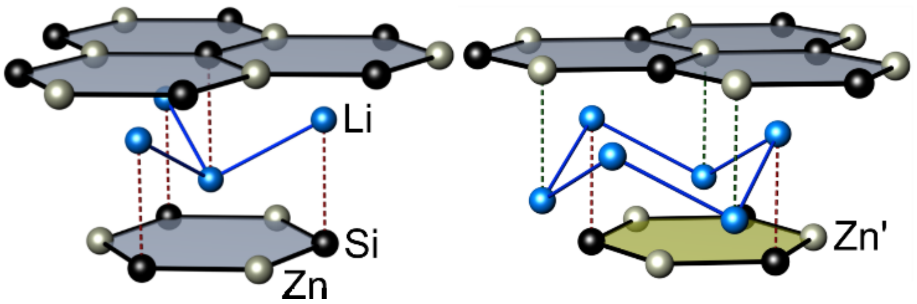

Figure S1. Local Li coordination environment before and after the Zn-Si layer shift. In the ideal structure (left), each Li atom has a nearest-neighbor Si atom along [001] direction. At the defect layer (right), this [001] neighbor is replaced by a Zn atom.

Table S7. Structure model of Li_2ZnSi in space group *R3m* (No. 160) with ABC packing of the ZnSi-layers (*a* = 4.25 Å; *c* = 12.34 Å). Optimization shows that the model is unstable.

Atom	Site	General coordinates		
Zn	3*a*	0	0	1/3
Si	3*a*	0	0	0
Li	3*a*	2/3	1/3	0.11
Li	3*a*	2/3	1/3	–0.11

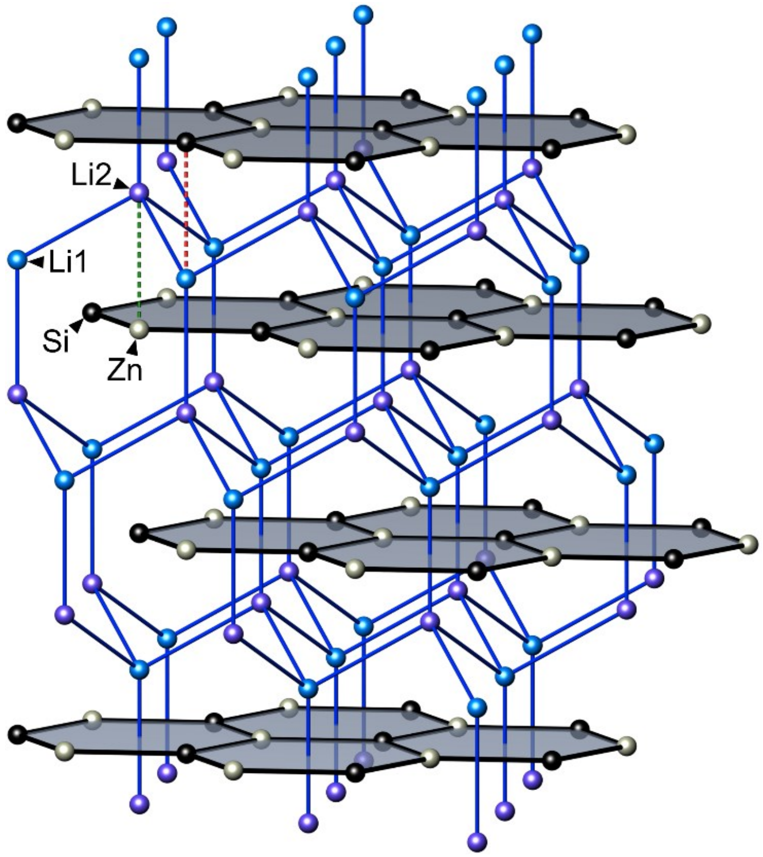

Figure S2. While the ideal structure of Li_2ZnSi features an AB stacking of the Zn-Si layers, ABC stacking is observed at defect layers. The image shows a structure model derived from the local coordination of the stacking faults (ABC stacking of the Zn-Si-layers) in the rhombohedral space group *R*3*m* (*a*′ = *a*, *c*′ = 3/2 *c*). The main difference from the ideal structure is that 50% of the Li–Si contacts along the [001] direction are replaced by Li–Zn contacts. In this configuration, the Li atoms adopt a diamond substructure. Unfortunately, calculations show that such this arrangement is thermodynamically unstable.

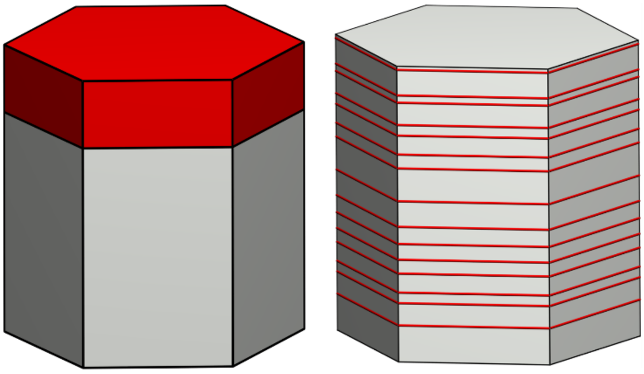

Figure S3: Alternative models for stacking faults: (Left) Single stacking fault creating a rotation twin vs. (Right) statistic distribution of stacking faults (split site model).

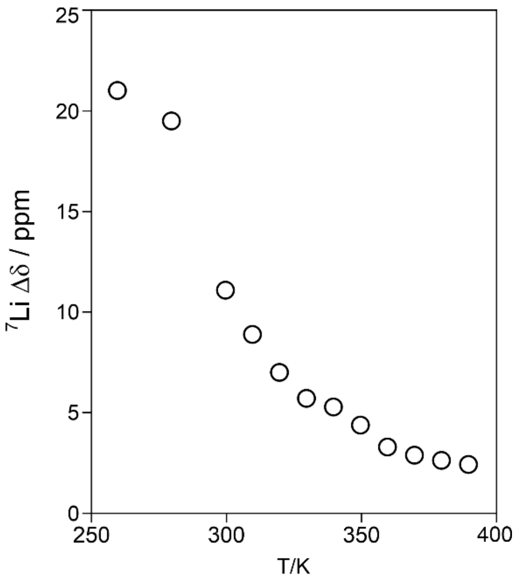

Figure S4: Linewidths of temperature-dependent ^{7}Li NMR spectra extracted from spectral fits.

Table S7. Optimized structure model for Li_2ZnSi in space group *P*3*m*1 (No. 156)

a = 4.25018 Å, *c* = 8.21234 Å. The Zn1 position was fixed to *z* = 0.25.

Atom	Site	General coordinates			Optimized coordinates		
Zn1	1*a*	0	0	*z*	0	0	**0.25**
Zn2	1*a*	0	0	*z*	0	0	0.7500001503
Si1	1b	1/3	2/3	*z*	1/3	2/3	0.2499986079
Si2	1c	2/3	1/3	*z*	2/3	1/3	0.7500014249
Li1	1b	1/3	2/3	*z*	1/3	2/3	0.9171214821
Li2	1c	2/3	1/3	*z*	2/3	1/3	0.4171470060
Li3	1c	2/3	1/3	*z*	2/3	1/3	0.0828521652
Li4	1b	1/3	2/3	*z*	1/3	2/3	0.582879174

Li positions based on experimental data are, e.g. *z* = 0.9176 (Li1) and *z* = 0.5824 (Li4).

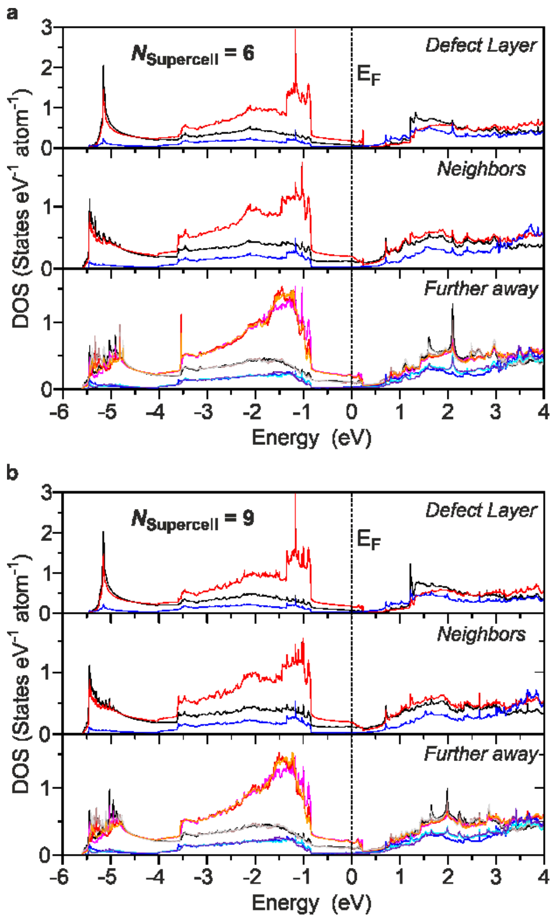

Figure S5. Atom-projected DOS computed for the 1 x 1 x 6 and 1 x 1 x 9 supercells. Black (and brown, gray) curves are for Zn, red (and magenta, orange) for Si, blue (and cyan, violet) for Li.

The computed defect formation energies remain remarkably constant as the supercell size is increased. The concentration-independent defect formation energy suggests that the energetic effects of the defect layer are highly localized. To determine if the atom-projected densities of states (pDOS) also corroborate this local nature of the defect, the electronic structures of 1 x 1 x 6 and 1 x 1 x 9 supercell models containing one defect layer are analyzed. The top panels of Fig.S5a and Fig.S5b show the pDOS of Zn and Si atoms in the defect layer, and of the Li atoms that have moved. In the crystal structure of Li_2ZnSi, the Zn-Si layers have mirror plane symmetry; therefore, the pDOS of the atoms in the layers that are equidistant from the defect layer are identical. Hence, in the other panels, pDOS of only one of them are shown. The middle panels display the contributions from the neighboring Zn-Si layers (± $c/2$ apart) and the associated Li atoms. In the bottom panel, the pDOS of Zn and Si atoms in the

layers that are c, $3c/2$ and $2c$ away from the defect layer are plotted. The pDOS of the Li atoms surrounding these layers are also shown. For both N_{sc} = 6 and N_{sc} = 9 cases, the pDOS of the neighboring atoms resemble those of the further away atoms, although it is clear that they are mainly of transitionary nature.
